# Some Common Mistakes in Gynecological Diagnosis*Philadelphia Medical Journal.

**Published:** 1898-05

**Authors:** J. C. Webster

**Affiliations:** M.D., F.R.C.P. Edin., of Montreal; Demonstrator of Gynecology, McGill University; Assistant Gynecologist, Royal Victoria Hospital, Montreal


### SOME COMMON MISTAKES IN GYNECOLOGICAL DIAGNOSIS.*

BY J. C. WEBSTER, M.D., F.R.C.P. EDIN., OF MONTREAL.

Demonstrator of Gynecology, McGill University; Assistant Gynecologist, Royal Victoria Hospital, Montreal.

The difficulty of establishing correct diagnoses in diseases of women is acknowledged by all who work at this specialty, generally, most readily acknowledged by those who have worked longest and most conscientiously. Owing to the nature of the subject it is not possible, in the great majority of medical schools, to give students as thorough a training in gynecology and obstetrics as that which can be given in the other branches of medicine. It is not surprising, therefore, that such a large proportion of men enter upon general practice feeling themselves unfit to cope with the special difficulties associated with the study of diseases peculiar to women. While it is safe to say that no one can obtain a mastery in this subject without undergoing a thorough training in hospitals where there is an abundance of clinical material, as well as skilled teachers, it is also true that a young practitioner, even in remote country districts, may, by constant care and attention, escape many of the dangers which arise from ignorance and may with a considerable degree of success carry out therapeutic measures.

The greatest difficulty in the establishment of accurate diagnosis in gynecology is that symptomatology and clinical history are indeterminate and, in the great majority of instances, can not be distinctly correlated with various lesions. Take, for example, the symptom most frequent in women's diseases, viz., pain. Owing to the peculiarities of the female constitution in abnormal states, it is with the greatest difficulty that the most skillful physician can truly estimate the place and significance of this symptom in the various cases which he considers. For, in regard to it, the following points must be kept in view:

1. The pain may be directly due to distinct pelvic lesions, sufficient in themselves to produce this symptom.
2. Pain may exist with minor degrees of pelvic trouble, insufficient in themselves to cause more than a small amount of suffering.
3. Pain may be a pelvic symptom in association with some condition which in itself can not directly produce the symptom.
4. It may be a prominent symptom in cases in which no local changes of any kind can be made out.

It is very evident, therefore, that other than local factors must be taken into account as explanatory of the subjective phenomena which comes under consideration. Of chief importance among these is the neuropathic state—neurosis, in the widest meaning of the word. This condition is related to the

*Philadelphia Medical Journal.

pelvis in various ways. In one set of cases, a local lesion, capable or not in itself of causing pain, may be the primary cause of development of a neurotic condition manifested by diverse phenomena. The more marked these become, the more is the pelvic pain intensified—a reactionary exhibition of the neurosis, as it were, on the seat of primary action. In another class of cases there may be a slight pelvic lesion causing very little discomfort.

A neurotic condition may be developed from causes foreign to the pelvis, and this may manifest itself in intense pain, referred by the patient to the pelvic lesion. In another set, the symptom of pelvic pain is developed as one of the phenomena of a widespread neuropathic state, there being no local lesion of any kind.

There is another interesting class in which the local symptom is practically the only neurotic feature in the patient. In some of these cases the condition is somewhat like that in which the possession of a "fixed idea" is characteristic. In others, it is of the nature of a "secondary reflex action," induced by a former continuity of habit, when there was an actual painful local lesion which has since been cured; the patient's nervous system has so registered the former habit that it is reproduced apart from all control of the cortical inhibitory centers. It may be of interest to refer to a number of cases studied in hospital practice, during a number of years, illustrative of some of the difficulties to which I allude.

I shall, in this paper, limit myself to certain disorders relating to the urinary tract.

It is not at all an uncommon occurrence that patients are sent to a gynecological department complaining of pain in the bladder, having been treated without success as cases of cystitis. The pain may be continuous, irregular, or felt only at times of micturition. The following case is worthy of notice:

Mrs. H., aged thirty-four, complains of pain in the bladder varying in intensity from time to time, with frequency of micturition. Her physician states that her temperature has been elevated and irregular for some weeks, that there has been pus in the urine, and that there is tenderness when the base of the bladder is palpated *per vaginam*. She has been fed on a milk diet and the bladder has been washed out daily with boracic lotion.

On making an abdomino-vaginal bimanual examination in this case, I found that the patient complained of tenderness in the bladder, but there was no thickening whatever to be made out. The viscus appeared to be perfectly normal.

Inspection of the interior of the bladder by Kelly's method demonstrated that the mucosa was perfectly healthy, with the exception of slight congestion around the right ureteric orifice. Palpation of the loins revealed an enlargement on the right side, and bacteriologic examination of the urine proved that it contained the *tubercle-bacillus*. Subsequent operation resulted in the removal of a tuberculous right kidney.

The mistake made in this case was due to the fact that the localization of the pain in the region of the bladder led to a centralization of the attention on that viscus alone. Now, it is very important to bear in mind that in some cases of kidney disease, *e. g.*, stone, tuberculosis, new growth, no pain may be felt for a considerable period in the seat of the disease, but only in the bladder. Sometimes in such cases the patient is sent to the hospital with the diagnosis of "stone in the bladder." I have also seen a case treated for some time as cystitis, on account of painful micturition, which proved to be an early symptom of locomotor ataxia. A similar mistake has been made in more advanced tabes when, besides the pain, frequency of micturition was also present. Fortunately, however, tabes is very uncommon in females, and such an error can but rarely occur.

I have seen a number of cases treated for some time as cystitis in which the pain was entirely neurotic in nature. Two of these were young unmarried women belonging to a rich and luxurious grade of society, whose indulgent physicians gratified their desire to have frequent digital examinations of the pelvis made for the purpose of studying the progress of supposed bladder-troubles. Both of these patients also found great relief in frequent irrigation of the bladder when it was performed by the physician. When, however, a stern nurse was put to this task, they experienced no longer any relief. In each of these cases hypnotism quickly led to a complete cure.

I remember another case of this kind which came under the care of a very prosaic and stern Scotch physician, after a year's treatment for cystitis. He assured her that he would cure her in one sitting. His cure consisted in chloroforming her and dilating the urethra. The interior of the bladder was found to be perfectly healthy. The patient had incontinence of urine and suffered real pain for some hours following the "operation," but her old trouble disappeared, and she was sent to the seaside under the care of a judicious nurse to enjoy a course of cold bathing.

In various pelvic affections, also, the most prominent symptom may be pain in the bladder, leading to a diagnosis of cystitis, the actual trouble being overlooked. As an example may be mentioned a case of a swelling in the broad ligament, in close relationship to the bladder. The swelling had never been found because attempts at a bimanual examination had caused excessive pain. The treatment employed was daily irrigation of the bladder with weak mercuric chlorid solution. When the patient was examined under an anesthetic, the swelling was found to be a chronic parametric abscess bulging into the fornix vaginæ. It was easily evacuated and the bladder symptoms immediately disappeared.

Similar errors may be made when tubal, ovarian, and other inflammatory affections exist. I recall an interesting case that I saw several years ago in a well-known German gynecological clinic. It was that of a Russian lady-doctor who was working in an operative course with myself. One day she did not appear with the rest of the foreigners. When I went into the operating room I was greatly surprised to find her on the table, chloroformed and ready for the knife. The operation consisted in the removal of a myoma, about the size of a hazel-nut, from the anterior uterine wall, near the attachment of the bladder. She had been suffering from vesical pain for several months, and had been under a course of treatment for cystitis at a Spa, without any improvement. After the operation she was completely relieved, and, in the course of a few weeks, again took her place among her fellow-workers in the clinic.

In rheumatic and gouty people very severe vesical pain may sometimes be found, and may be diagnosticated and treated as cystitis, though no bladder-changes can be made out on careful examination. In such cases improvement often follows proper suitable treatment, but sometimes the symptom is due to a renal calculus.

Cases are also not infrequently found in which disease of the bladder is treated as something else, the real trouble being overlooked. Thus, I have known carcinoma of the bladder treated as kidney-disease, because of the absence of vesical pain. It is certainly a mistake to believe that this condition must necessarily be associated with pain. In hard carcinoma of the vesical wall this is indeed an early symptom, but in soft carcinoma there may be no pain whatever, until cystitis develops, or until the growth is so large as to obstruct the urethra. The mistake is also made of treating this malignant condition as one of simple cystitis. Sometimes, also, the chief troubles in this disease are related to the rectum, leading to the formation of an incorrect diagnosis. In one case the pain was related to the coccyx, and the patient was subjected to an operation for the removal of that structure, under the belief that she was suffering from coccygodynia. Tuberculous ulceration of the bladder has been frequently treated as chronic cystitis until cystoscopic and bacteriologic examination revealed the characteristic signs.

Several years ago I had under my care an interesting case of a woman who had been under medical treatment for hematuria of supposed kidney-origin. It was thought that slowly progressing malignant renal disease existed. Repeated bimanual examination of the pelvis had been made, but nothing abnormal had ever been noted in connection with the bladder. The patient was very anemic, but there was no vesical pain, and only occasionally frequency of micturition. The amount of blood in the urine had never been great, and varied from time to time.

On dilating the urethra I saw through the speculum nearly a dozen small nodules at the base of the bladder, the largest being less than the size of half a pea. Around several of them spicules of lime were recognized, and the mucosa of the whole base of the bladder was congested. I afterwards split the vesico-vaginal septum from the urethra to the cervix uteri and removed the nodules, which were of a soft, warty nature, cauterizing their areas of attachment. The bladder was then closed and the patient recovered, being afterwards free from hematuria.—*Atlantic Medical Weekly.*

